# The roles of SARP family regulators involved in secondary metabolism in *Streptomyces*

**DOI:** 10.3389/fmicb.2024.1368809

**Published:** 2024-03-14

**Authors:** Yusi Yan, Haiyang Xia

**Affiliations:** ^1^Institute of Biopharmaceuticals, Taizhou University, Taizhou, China; ^2^NHC Key Lab of Reproduction Regulation (Shanghai Institute for Biomedical and Pharmaceutical Technologies), School of Public Health, Fudan University, Shanghai, China

**Keywords:** *Streptomyces*, SARP family regulator, biosynthetic gene cluster, secondary metabolism, biotechnological application

## Abstract

*Streptomyces* species are best known for their ability to produce abundant secondary metabolites with versatile bioactivities and industrial importance. These metabolites are usually biosynthesized through metabolic pathways encoded by cluster-situated genes. These genes are also known as biosynthetic gene clusters (BGCs) of secondary metabolites. The expression of BGCs is intricately controlled by pyramidal transcriptional regulatory cascades, which include various regulators. *Streptomyces* antibiotic regulatory proteins (SARPs), a genus-specific family of regulators, are widely distributed and play important roles in regulating the biosynthesis of secondary metabolites in *Streptomyces*. Over the past decade, the biological functions of SARPs have been extensively investigated. Here, we summarized the recent advances in characterizing the roles of SARPs involved in *Streptomyces* secondary metabolism from the following three aspects. First, the classification and domain organization of SARPs were summarized according to their size variation. Second, we presented a detailed description of the regulatory mechanisms and modes of action of SARPs involved in secondary metabolism. Finally, the biotechnological application of SARPs was illustrated by improving the production of target secondary metabolites and discovering novel bioactive natural products. This review will help researchers to comprehensively understand the roles of SARPs in secondary metabolite biosynthesis in *Streptomyces*, which will contribute to building a solid foundation for their future application in synthetic biology.

## Introduction

*Streptomyces* is a group of actinobacteria with a high GC content in their genomic DNA and complex morphological differentiation and life cycles (Hopwood, 2019). These bacteria are best known for their extraordinary ability to produce a multitude of bioactive secondary metabolites, such as antibiotics, insecticides and immunosuppressants, which make important contributions to clinical, medicinal and industrial fields (Hutchings et al., 2019; Jose et al., 2021; Alam et al., 2022). Typically, secondary metabolites are biosynthesized through secondary metabolic pathways that are encoded by biosynthetic gene clusters (BGCs) (Sharma et al., 2021). The morphological differentiation and expression of BGCs are intricately and stringently governed by pyramidal transcriptional regulatory cascades formed by large numbers of regulators and various signals (Wei et al., 2018; Xia et al., 2020). These regulators can be hierarchically grouped into global/pleiotropic and pathway-specific regulators according to their target genes and pathways. The bottom level consists of pathway-specific regulators (PSRs), which affect the production of cognate secondary metabolites by directly controlling the transcription of biosynthetic genes. PSR-encoding genes are usually located in BGCs and are also called cluster-situated regulators (CSRs) (Lu et al., 2017; Wei et al., 2018). Global/pleiotropic regulators function at higher levels. The genes that encode these regulators are usually located outside the BGCs, and they also regulate the expression of CSR-encoding genes, the biosynthesis of multiple secondary metabolites and/or morphological differentiation (Liu et al., 2013; Niu et al., 2016).

*Streptomyces* antibiotic regulatory proteins (SARPs) are a specific family of regulators exclusively found in actinobacteria, especially in streptomycetes (Bibb, 2005; Krause et al., 2020). Generally, SARP-encoding genes are widely present in many different types of BGCs and play indispensable roles in controlling the biosynthesis of secondary metabolites in *Streptomyces*. To the best of our knowledge, only one review has summarized the roles of SARPs in secondary metabolite biosynthesis in *Streptomyces coelicolor* (Liu et al., 2013). Over the past decade, many SARPs have been characterized in different *Streptomyces* strains, and their roles in secondary metabolite biosynthesis are more diverse than previously reported (Li et al., 2018; Yang et al., 2019; Yan et al., 2022). This review highlights recent findings on the mechanism by which SARPs control secondary metabolism in *Streptomyces*. A comprehensive understanding of the regulatory mechanism of SARPs on secondary metabolite biosynthesis will facilitate their biotechnological application to improve the production of important secondary metabolites and discover novel bioactive natural products in the near future.

## The functional domain organization and classification of SARPs

**Figure 1:**
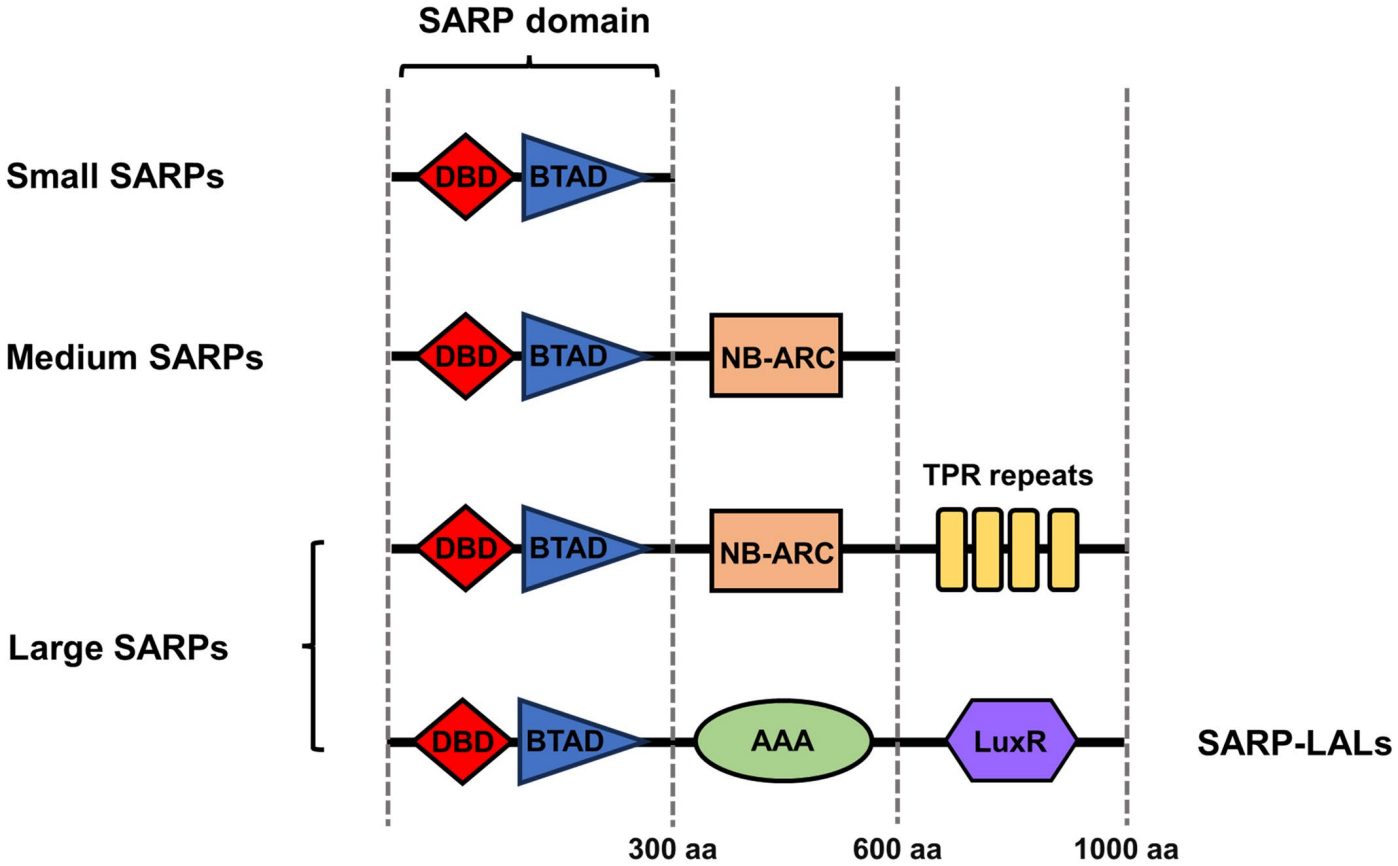
Classification and domain organization of SARP family regulators. The red rhomb indicates the DNA binding domain (DBD). The blue triangle indicates the bacterial transcriptional activation domain (BTAD). The orange rectangle indicates the NB-ARC domain. The yellow rectangles indicate the TRP repeats. The green oval indicates the AAA domain. The violet hexagon indicates the partially homologous LuxR domain in SARP-LAL. The architectures indicate only domains, not lengths.

SARPs have highly variable lengths and functional domain organizations. By their size and domain organization pattern, SARPs can be divided into three groups: small SARPs, medium SARPs and large SARPs (Figure 1). Small SARPs are approximately 300 residues in length and contain only an N-terminal DNA-binding domain (DBD) and a C-terminal bacterial transcriptional activation domain (BTAD); these include RedD for undecylprodigiosin (RED) biosynthesis, ActII-ORF4 for actinorhodin (ACT) biosynthesis and CpkN for coelimycin biosynthesis in *S. coelicolor* (Bednarz et al., 2021). Additionally, these two domains are typical SARP domains in which the DBD binds to repeated DNA motifs and the adjacent BTAD is supposed to initiate the transcription of target BGCs by recruiting RNA polymerase (RNAP) (Liu et al., 2013).

Medium SARPs, such as CdaR for calcium-dependent antibiotic (CDA) biosynthesis and CpkO for coelimycin biosynthesis in *S. coelicolor* and FdmR1 for fredericamycin biosynthesis in *Streptomyces griseus,* usually contain approximately 600 amino acid residues and consist of a SARP domain and an NB-ARC domain (Chen et al., 2008; Bednarz et al., 2021). The NB-ARC domain is a highly conserved nucleotide-binding domain shared with APAF-1, various R proteins and CED-4. The NB-ARC domain works as a signaling motif found in eukaryotes and bacteria and is characteristic of the AAA domain superfamily, which is widely considered to act as a molecular switch to the cycle between ADP (repressed) and ATP (active)-bound forms (Steele et al., 2019).

Large SARPs contain approximately 1,000 residues and consist of an N-terminal SARP domain, a central NB-ARC domain and a conserved C-terminal tetratricopeptide repeat (TPR) domain; examples include RslR3 for rishirilide biosynthesis in *Streptomyces bottropensis* and PolY for polyoxin biosynthesis in *Streptomyces cacaoi* subsp. *asoensis* (Li et al., 2010; Tsypik et al., 2021). The TPR domain usually mediates protein–protein interactions and the assembly of multiprotein complex mediators (D’Andrea and Regan, 2003).

In addition, several large SARPs have specific domain architectures, which include an N-terminal SARP domain and half of a C-terminal that is homologous to guanylate cyclases and LAL regulators (large ATP-binding regulators of the LuxR family) (Li et al., 2022). The C-terminal half includes the ATP/GTP binding domain that is characteristic of these protein families but lacks the signature sequence at the N-terminus of guanylate cyclases or the LuxR-type helix-turn-helix (HTH) motif for DNA binding present at the C-terminus of LAL regulators (Barreales et al., 2018). Therefore, these special kinds of large SARPs are also called SARP-LALs and include SanG for nikkomycin biosynthesis in *Streptomyces ansochromogenes*, FilR for filipin biosynthesis in *Streptomyces filipinensis* and PimR for pimaricin biosynthesis in *Streptomyces natalensis* (He et al., 2010; Santos-Aberturas et al., 2012; Barreales et al., 2018).

In this review, 25 representative *Streptomyces* strains were chosen to illustrate the distribution of SARPs. The genomic sequences of these strains from GenBank were analyzed via the Predicted Prokaryotic Regulatory Proteins (P2RP) webserver and antiSMASH webserver for the distribution of SARPs and BGCs, respectively (Barakat et al., 2013; Blin et al., 2024) (Table 1). Generally, more SARPs exist in strains with larger genomes and more BGCs. Additionally, more than half of the strains contained all the kinds of SARPs mentioned above. In particular, homologs of AfsR, a special kind of large SARP, are distributed among all the tested *Streptomyces* species.

**Table 1:** The distribution of putative SARP family regulators in *Streptomyces* species.

Organism	Genome size Mb	BGCs	SARP family regulators					
			Total	Small	Medium	Large	SARP-LAL	Homologs of AfsR
*S. bingchenggensis* BCW-1	11.94	49	27	5	6	10	5	SBI_06321
*S. spectabilis* ATCC 27465	9.81	43	21	2	3	12	3	CP982_19265
*S. violaceusniger* Tü 4113	11.14	43	18	5	3	8	1	Strvi_0998
*S. hygroscopicus* subsp. *jinggangensis* 5008	10.38	38	18	3	5	6	3	SHJG_4714
*S. tsukubaensis* NRRL 18488	8.02	35	17	4	5	7	0	B7R87_RS12770
*S. griseus* subsp. *griseus* NBRC 13350	8.54	38	16	2	5	7	1	SGR_3012
*S. clavuligerus* ATCC 27064	8.56	35	15	3	4	5	2	SCLAV_RS16135
*S. rochei* 7434AN4	8.37	32	15	5	1	5	3	F1617_RS28730
*S. davaonensis* JCM 4913	9.56	33	13	3	2	4	3	BN159_5015
*S. clavuligerus* F613-1	7.59	34	12	2	4	3	2	BB341_RS11760
*S. collinus* Tü 365	8.38	34	11	4	1	4	1	B446_16915
*S. fulvissimus* DSM 40593	7.91	34	11	0	4	5	1	SFUL_4345
*S. chattanoogensis* NRRL ISP-5002	9.13	51	11	1	2	5	2	ADL29_RS21155
*S. scabiei* 87.22	10.15	34	10	1	3	3	2	SCAB51941
*S. venezuelae* ATCC 10712	8.23	30	10	2	0	6	0	SVEN_3095
*S. natalensis* ATCC 27448	8.65	34	9	0	4	4	0	SNA_RS37840
*S. avermitilis* MA-4680	9.12	36	9	3	0	3	2	SAVERM_3804
*S. coelicolor* A3(2)	8.67	27	8	3	2	2	0	SCO4426
*S. albus* DSM 41398	8.39	36	8	2	3	0	2	SLNWT_RS19955
*S. cattleya* NRRL 8057	8.09	15	8	2	1	3	1	SCAT_2348
*S. peucetius* ATCC27952	8.02	21	7	2	2	2	0	CGZ69_15445
*S. cacaoi* OABC16	8.58	43	7	2	1	1	2	F1617_RS28730
*S. viridochromogenes* DSM 40736	8.65	32	6	2	3	0	0	SSQG_03410
*S. albidoflavus* J1074	6.84	22	4	1	2	0	0	XNR_2231
*S. fradiae* ATCC 10745	6.73	29	4	0	0	3	0	CP974_12720

Genomic data from The National Center for Biotechnology Information (NCBI) website (https://www.ncbi.nlm.nih.gov) were analyzed via the Predicted Prokaryotic Regulatory Proteins (P2RP) webserver (http://www.p2rp.org) and the antiSMASH webserver (https://antismash-db.secondarymetabolites.org).

## The regulatory function of SARPs in secondary metabolism

### The SARP global regulator AfsR

To date, AfsR has been identified as the single global regulator of the SARP family and has been extensively studied in *S. coelicolor* and *S. griseus*. AfsR positively regulates the biosynthesis of ACT, RED and CDA by directly activating the expression of the adjacent gene *afsS*, which encodes a small sigma factor-like protein. In turn, the synthesis of AfsS activates the transcription of genes encoding pathway-specific activators, which control the transcription of the ACT, RED and CDA BGCs in an as yet unknown way (Umeyama et al., 2002; Liu et al., 2013).

AfsR is phosphorylated at serine/threonine residues by the AfsK kinase (Matsumoto et al., 1994; Jin et al., 2011). The phosphorylation of AfsR significantly increases its binding affinity to the *afsS* promoter and therefore enhances the yields of ACT, RED and CDA (Martín and Liras, 2019). Notably, the autophosphorylating activity of AfsK is inhibited by KbpA, whose encoding gene is located upstream of *afsK* in both *S. coelicolor* and *S. griseus*. KbpA binds to unphosphorylated AfsK to inhibit its autophosphorylation at serine/threonine residues. Therefore, KbpA acts as a negative regulator in the AfsK–AfsR phosphorylation cascade, which indirectly decreases secondary metabolism in *S. coelicolor* and sporulation in *S. griseus* (Umeyama and Horinouchi, 2001; Sawai et al., 2004). In addition, the C-terminus of AfsK is likely involved in binding *S*-adenosyl-L-methionine (SAM) in *S. coelicolor*. SAM activates actinorhodin biosynthesis by increasing the autophosphorylation of AfsK (Lee et al., 2007; Jin et al., 2011). Moreover, two other serine/threonine kinases, AfsL and PkaG, that can phosphorylate AfsR *in vitro* have been identified (Sawai et al., 2004; Liu et al., 2013; Martín et al., 2021). This finding suggested that AfsR works as a signal integrator of signals that are sensed by multiple serine/threonine kinases (Horinouchi, 2003).

The binding site of AfsR at the *afsS* promoter consists of two direct repeats (CGTT(T/C)ATCGNN), which are also recognized by another global regulator, PhoP (Santos-Beneit et al., 2009). The two-component system PhoR–PhoP controls global phosphate metabolism and secondary metabolite biosynthesis in *Streptomyces*. The response regulator PhoP binds to the *afsS* promoter in a region overlapping with the AfsR binding sequence, and there is the binding competition between these two regulators. Additionally, AfsR binds to other PhoP-regulated promoters, including those of *pstS* (a component of the phosphate transport system), *phoRP* (encoding the two-component system itself) and *glnR* (encoding the regulator of nitrogen metabolism) (Santos-Beneit et al., 2012; Van der Heul et al., 2018). In summary, AfsR is involved in regulating primary and secondary metabolism through cross-talk with PhoP and GlnR.

In addition, AfsR homologs from other *Streptomyces* species have also been shown to be involved in morphological development and/or secondary metabolite biosynthesis. In *S. griseus*, AfsR is also required for morphological development but is not directly needed to produce streptomycin or A-factors (Umeyama et al., 1999). In *Streptomyces roseosporus*, AfsR works as an activator of daptomycin production but plays a negative role in development (Yan et al., 2020). Moreover, in *Streptomyces venezuelae*, *Streptomyces peucetius*, *Streptomyces acidiscabies* and *Streptomyces lomondensis*, AfsR homologs activate the biosynthesis of various secondary metabolites (Maharjan et al., 2009; Kim et al., 2012; Wang et al., 2015) (Table 2). However, AfsR represses the production of pristinamycin I but activates the production of pristinamycin II in *Streptomyces pristinaespiralis* (Jin et al., 2021). Notably, AfsR of *S. peucetius* is named AfsR-sp., and its encoded by the *afsR*-sp. gene. Overexpression of *afsR*-sp. under the control of a strong promoter led to improved doxorubicin production in *S. peucetius* (Maharjan et al., 2009). A similar strategy has also been used to overproduce actinorhodin in *S. lividans*, clavulanic acid in *S. clavuligerus* and pikromycin in *S. venezuelae* by overexpressing *afsR*-sp. from *S. peucetius* (Parajuli et al., 2005) (Table 2).

**Table 2:** Identified homologs of AfsR involved in the biosynthesis of secondary metabolites in *Streptomyces* species.

Regulators	Strains	Secondary metabolites	BCG type	Effect	Increased yield	References
AfsR	*S. coelicolor* A3(2)	Undecylprodigiosin	Type I PKS	Activator	/	Liu et al. (2013)
		Calcium-dependent antibiotic	NRPS			
		Actinorhodin	Type II PKS			
AfsR-sv	*S. venezuelae* ATCC 15439	Pikromycin	Type I PKS	Activator	/	Maharjan et al. (2009)
AfsR-sp	*S. peucetius* ATCC 27952	Doxorubicin	Type II PKS	Activator	400% doxorubicin 260% actinorhodin* 150% clavulanic acid* 260% pikromycin*	Parajuli et al. (2005)
AfsR	*S. acidiscabies* ATCC 49003	WS5995B	Type II PKS	Activator	/	Kim et al. (2012)
		Thaxtomin	NRPS			
AfsR	*S. pristinaespiralis* Pr11	Pristinamycin I	NRPS	Repressor	/	Jin et al. (2021)
		Pristinamycin II	NRPS/PKS	Activator		
AfsR	*S. roseosporus* NRRL11379	Daptomycin	NRPS	Activator	120%	Yan et al. (2020)
AfsR	*S. lomondensis* S015	Lomofungin	NRPS	Activator	250%	Wang et al. (2015)

The AfsR of *S. venezuelae* is named AfsR-sv and regulates pikromycin biosynthesis. The AfsR of *S. peucetius* is named AfsR-sp. and regulates doxorubicin biosynthesis.

*Overexpression of *afsR*-sp. in *S. lividans* improved the production of actinorhodin. Overexpression of *afsR*-sp. in *S. clavuligerus* led to increased clavulanic acid production. Overexpression of *afsR*-sp. in *S. venezuelae* resulted in enhanced production of pikromycin.

### The various regulatory modes of SARPs

Typically, SARP-encoding genes are located in various BGCs that encode polyketides (Type I or Type II PKS), nonribosomally synthesized peptides (NRPS), hybrid polyketide peptide (NRPS/PKS) compounds, nucleosides, thiopeptides and terpenoids (Krause et al., 2020) (Table 3). Most of them act as pathway-specific activators of secondary metabolite biosynthesis by directly controlling the transcription of their cognate BGCs; for example, ChIF2 is involved in chlorothricin biosynthesis in *Streptomyces antibioticus*, NigR is involved in nigericin biosynthesis in *Streptomyces malaysiensis,* and PieR is involved in piericidin biosynthesis in *Streptomyces piomogeues* var. Hangzhouwanensis (Li et al., 2019, 2020; Wei et al., 2022) (Table 3). Notably, the medium-sized SARP Atr32 has been identified to negatively affect atratumycin biosynthesis in *Streptomyces atratus*. The function of Atr32 was distinct from that described in previous studies, but the underlying mechanism has not been elucidated (Yang et al., 2019). Moreover, multiple SARP-encoding genes located in the same BGC often form a hierarchical cascade of regulation to control the biosynthesis of cognate secondary metabolites. For example, two SARP activators, CpkO and CpkN, are necessary for coelimycin biosynthesis in *S. coelicolor*, in which CpkO activates the transcription of *cpkN* (Bednarz et al., 2021). In *S. cacaoi* subsp. *asoensis*, PolY positively controls polyoxin biosynthesis by directly activating the transcription of *polR,* which encodes another SARP (Li et al., 2010). Three SARPs, RslR1, RslR2, and RslR3, are positive regulators of rishirilide biosynthesis in *S. bottropensis*. RslR3 directly activates the transcription of *rslR2*, while RslR1 represses the transcription of *rslR2* (Tsypik et al., 2021).

**Table 3:** Identified SARP family regulators involved in the biosynthesis of secondary metabolites in *Streptomyces* species.

Regulators	Strains	Secondary metabolites	The type of BGCs	Effect	Increased yield	References
**Large SARPs**						
PolY	*S. cacaoi* subsp*. asoensis* AS4.1602	Polyoxin	Nucleoside	Activator	/	Li et al. (2010)
RslR3	*S. bottropensis*	Rishirilide	Type II PKS	Activator	370%	Tsypik et al. (2021)
Orf4	*S. echinatus* Tü 303	Aranciamycin	Type II PKS	Activator	/	Luzhetskyy et al. (2007)
**SARP-LALs**						
FilR	*S. filipinensis* DSM 40112	Filipin	Type I PKS	Activator	/	Barreales et al. (2018)
PimR	*S. natalensis* ATCC 27448	Pimaricin	Type I PKS	Activator	/	
PteR	*S. avermitilis* NRRL 8165	Filipin	Type I PKS	Activator	/	
ScnRI	*S. chattanoogensis* L10	Natamycin	Type I PKS	Activator	/	
PnR2	*S. platensis* SAM-0654	Phoslactomycin	Type I PKS	Activator	180%	Chen et al. (2012)
PolR	*S. cacaoi* subsp*. asoensis* AS4.1602	Polyoxin	Nucleoside	Activator	300%	Li et al. (2009)
SanG	*S. ansochromogenes* 7100	Nikkomycin	Nucleoside	Activator	200%	Xia et al. (2020)
**Medium SARPs**						
BafG	*S. lohii* ATCC BAA-1276	Bafilomycin	Type I PKS	Activator	150%	Li et al. (2021)
CpkO	*S. coelicolor* A3(2)	Coelimycin	Type I PKS	Activator	/	Bednarz et al. (2021)
BenR	*Streptomyces* sp. A2991200	Benastatin	Type II PKS	Activator	/	Xu et al. (2007)
FdmR1	*S. griseus* ATCC 49344	Fredericamycin	Type II PKS	Activator	560%	Chen et al. (2008)
SnoA	*S. nogalater* ATCC 27451	Nogalamycin	Type II PKS	Activator	/	Torkkell et al. (1997)
SCAB1371	*S. scabies* 87.22	Pyochelin	NRPS	Activator	/	Seipke et al. (2011)
CdaR	*S. coelicolor* A3(2)	Calcium-dependent antibiotic	NRPS	Activator	/	Liu et al. (2013)
Atr32	*S. atratus* SCSIO ZH16NS-80S	Atratumycin	NRPS	Repressor	/	Yang et al. (2019)
**Small SARPs**						
ArpRI	*S. argillaceus* ATCC 12956	Argimycins P	Type I PKS	Activator	94%	Ye et al. (2017)
AsuR5	*S. nodosus* subsp*. asukaensis* ATCC 29757	Asukamycin	Type I PKS	Activator	/	Xie P. et al. (2012)
ChIF2	*S. antibioticus* DSM 40725	Chlorothricin	Type I PKS	Activator	840%*	Li et al. (2020)
CpkN	*S. coelicolor* A3(2)	Coelimycin	Type I PKS	Activator	/	Chen et al. (2008)
MilR3/KelR	*S. bingchenggensis* TMB	Milbemycin Nanchangmycin	Type I PKS	Activator Repressor	138% 4,500%*	Wang et al. (2022) Yan et al. (2024)
MonRI	*S. cinnamonensis* ST021	Monensin	Type I PKS	Activator	/	Tang et al. (2017)
NanR1 NanR2	*S. nanchanggensis* NS3226	Nanchangmycin	Type I PKS	Activator	~300%	Yu et al. (2012)
NigR	*S. malaysiensis* F913	Nigericin	Type I PKS	Activator	168%	Wei et al. (2022)
RedD	*S. coelicolor* A3(2)	Undecylprodigiosins	Type I PKS	Activator	550%	Liu et al. (2013)
VmsR VmsS	*S. virginiae* MAFF 10-06014	Virginiamycin	Type I PKS	Activator	/	Kawachi et al. (2000)
TylS	*S. fradiae* T59235	Tylosin	Type I PKS	Activator	400%	Stratigopoulos et al. (2004)
ActII-Orf4	*S. coelicolor* A3(2)	Actinorhodin	Type II PKS	Activator	300%	Sohoni et al. (2014)
Alb45	*S. chrestomyceticus* BCC 24770	Albofungin	Type II PKS	Activator	130%	She et al. (2022)
AlpV	*S. ambofaciens* ATCC 23877	Alpomycin	Type II PKS	Activator	/	Lu et al. (2017)
Aur1PR3 Aur1PR4	*S. aureofaciens* CCM 3239	Auricin	Type II PKS	Activator	/	Kormanec et al. (2014)
DnrI	*S. peucetius* ATCC 29050	Daunorubicin	Type II PKS	Activator	/	Prija et al. (2017)
MilR3/KelR	*S. bingchenggensis* TMB	Yellow compound	Type II PKS	Activator	128%	Yan et al. (2022)
MtmR	*S. argillaceus* ATCC 12956	Mithramycin	Type II PKS	Activator	~155%	Flórez et al. (2015)
OtcR	*S. rimosus* M4018	Oxytetracycline	Type II PKS	Activator	649%	Yin et al. (2015)
RslR1 RslR2	*S. bottropensis*	Rishirilide	Type II PKS	Activator	400%	Tsypik et al. (2021)
SrcmRI	*S. roseiscleroticus* ATCC 53903	Chromomycin	Type II PKS	Activator	750%*	Sun et al. (2018)
Txn9	*S. bottropensis* NRRL 12051	Trioxacarcin	Type II PKS	Activator	/	Lu et al. (2017)
PieR	*S. piomogeues var. Hangzhouwanensis*	Piericidin A1	PKS	Activator	230%	Li et al. (2019)
BulY BulZ	*S. tsukubaensis* NRRL18488	Tacrolimus	NRPS/PKS	Activator	136% 67.4%*	Ma et al. (2018)
CcaR	*S. clavuligerus* ATCC 27064	Clavulanic acid Cephamycin	NRPS/PKS	Activator	200% ~ 300% 200% ~ 300%	López-Agudelo et al. (2021)
PapR1 PapR2 PapR4	*S. pristinaespiralis* Pr11	Pristinamycin	NRPS/PKS	Activator	100% 100% /	Mast et al. (2015)
SrrY SrrZ	*S. rochei* 7434AN4	Lankacidin and Lankamycin	NRPS/PKS	Activator	/	Suzuki et al. (2010)
FarR3 FarR4	*S. lavendulae* FRI-5	Indigoidine	NRPS	Activator	/	Kurniawan et al. (2014)
ThnU	*S. cattleya* NRRL8057	Cephamycin C	NRPS	Activator	/	Rodríguez et al. (2011)
SgvR2 SgvR3	*S. griseoviridis* NRRL 2427	Griseoviridin and Viridogrisein	NRPS	Activator	/	Xie Y. et al. (2012)
Orf22	*S. fungicidicus* ATCC 31731	Enduracidin	NRPS	Activator	366%	Chen et al. (2019)
VlmI	*S. viridifaciens* MG456-hF10	Valanimycin	NRPS	Activator	/	Garg and Parry (2010)
NosP	*S. actuosus* ATCC25421	Nosiheptide	Thiopeptide	Activator	120%	Li et al. (2018)
PlaR1	*Streptomyces* sp. Tü 6071	Phenalinolactone	Terpenoid	Activator	/	Dürr et al. (2006)

^*^ An improvement in the production of chlorothricin was achieved by coexpressing *chlF2* and its cotranscribed type II thioesterase-encoding gene *chlK*.

An improvement in the production of nanchangmycin was achieved by coexpressing *nanR1* and *nanR2*.

An increase in the production of chromomycin was achieved by overexpressing *srcmRI* and disrupting the PadR-like repressor-encoding gene *srcmRII*.

An increase in the production of tacrolimus was achieved by coexpressing *bulZ* and the GBL synthetase-encoding gene *bulS2*.

A few small SARP-encoding genes are located within the γ-butyrolactone (GBL) regulatory gene cluster and are involved in the GBL signaling pathway to control the biosynthesis of secondary metabolites (Table 3). In *Streptomyces tsukubaensis*, BulZ and BulY, located in the GBL region, were reported to positively regulate tacrolimus production (Ma et al., 2018). In another study, SrrY not only played a central role in the GBL signaling pathway but also activated lankamycin biosynthesis by directly regulating the transcription of *srrZ* in *Streptomyces rochei* (Suzuki et al., 2010). However, FarR3 and FarR4 have distinct effects on secondary metabolism in *Streptomyces lavendulae*, where FarR4 negatively controls GBL and indigoidine biosynthesis, but FarR3 positively regulates indigoidine biosynthesis (Kurniawan et al., 2014). Until now the detailed description of regulatory mode of FarR4 has remained unclear.

Notably, several small SARPs have also been reported to be pleiotropic regulators that control the biosynthesis of different secondary metabolites. CcaR from *Streptomyces clavuligerus*, which is encoded by *ccaR* located within the cephamycin BGC, acts as an activator of both cephamycin and adjacent clavulanic acid BGCs (López-Agudelo et al., 2021). In *Streptomyces binchenggensis*, *milR3*/*kelR* is located in a type II PKS BGC that is responsible for producing the yellow compound. The milbemycin BGC is far from the type II PKS BGC in the genome of *S. bingchenggensis*, but MilR3/KelR has been shown to coactivate the biosynthesis of milbemycin and the yellow compound (Wang et al., 2022; Yan et al., 2022). In addition, *S. bingchenggensis* can also produce nanchangmycin. NanR4, which is encoded by *nanR4* located in the nanchangmycin BGC, is a specific repressor of nanchangmycin biosynthesis. MilR3/KelR has been proven to inhibit nanchangmycin biosynthesis by activating the transcription of *nanR4* (Yan et al., 2024). Therefore, the small SARP MilR3/KelR can coregulate the biosynthesis of three secondary metabolites in *S. bingchenggensis*.

Recently, it has been reported that SARPs regulate the production of secondary metabolites by sensing final products and intermediates. As the only CSR related to nosiheptide (NOS) production in *Streptomyces actuosus*, NosP directly activates the transcription of biosynthetic genes by binding to the intergenic sequence in the NOS BGC. Furthermore, the DNA binding activity of NosP is modulated by NOS and its intermediate NOS-AC (Li et al., 2018). Moreover, NocP, the homolog of NosP in *Nocardia* sp., regulates the biosynthesis of structurally related nocathiacin I in a similar pattern (Li et al., 2018).

### The regulatory mechanisms of SARPs

SARPs usually activate the transcription of BGCs by binding to upstream regions of their target genes. Generally, heptameric direct repeats separated by spacers of 4–15 nucleotides are SARP-binding sequences that overlap the-35 region of the promoters of target genes (Liu et al., 2013). Moreover, the number of heptameric repeats in the SARP-binding site varies depending on the different SARPs. Some SARP-binding sites contain three heptameric repeats, such as DnrI-binding site in the upstream region of *dnrD* in *Streptomyces peucetius*, ActII-ORF4-binding sites in the upstream regions of *actII*-*ORF1* and *actVI*-*ORF1* in *S. coelicolor*, VlmI-binding sites in the upstream regions of *vlmJ* and *vlmA*-*vlmH* in *Streptomyces viridifaciens*, and FdmR1-binding site in the upstream region of *fdmD* in *S. griseus* (Tang et al., 1996; Chen et al., 2008; Garg and Parry, 2010; Liu et al., 2013). Other species have two obvious heptamers, including the SanG-binding site located between *sanO* and *sanN* in *S. ansochromogenes*, the PolR-binding site located between *polB* and *polC* in *S. cacaoi* subsp*. asoensis*, the CcaR-binding sites in the upstream regions of *cmcI* and *ceaS2-II* in *S. clavuligerus* and the PieR-binding site in the upstream region of *pieR* in *S. piomogeues* (Tahlan et al., 2004; Li et al., 2009, 2019; He et al., 2010).

Notably, the SARP-LALs involved in polyene macrolide biosynthesis work via a special mechanism different from that of other SARPs. The *pimR* gene, which encodes the SARP-LAL regulator, and the *pimM* gene, which encodes the PAS-LuxR regulator, are located in the pimaricin BGC in *S. natalensis*. PimR binds the promoter of *pimM* and activates its transcription, and in turn, PimM activates the transcription of biosynthetic genes in the pimaricin BGC (Santos-Aberturas et al., 2012; Li et al., 2022). The PimR-binding site contains three heptameric direct repeats separated by four nucleotide spacers that do not overlap the-35 promoter box (Tanaka et al., 2007; Santos-Aberturas et al., 2012). Additionally, PimR can bind a secondary operator with only two direct repeats separated by three-nucleotide spacers, forming 10-bp repeating units instead of the classical 11-bp SARP-binding sites (Wietzorrek and Bibb, 1997; Santos-Aberturas et al., 2012). Notably, the binding sequence of PimR is entirely conserved in the intergenic region between *scnRII* and *scnRI* in the natamycin BGC of *Streptomyces chattanoogensis* and between *pteF* and *pteR* in *Streptomyces avermitilis* and between *filF* and *filR* in *S. filipinensis*, which are the corresponding counterparts in the filipin BGC (Barreales et al., 2018). PimR, ScnRI, PteR and FilR are all SARP-LALs involved in polyene macrolide biosynthesis. It is thus likely that the hierarchical relationship between PimR and PimM is conserved in other polyene regulatory pathways. Interestingly, the consensus heptamer of PimR is also found in the binding site of SARP-LALs involved in peptidyl nucleoside biosynthesis, including SanG and PolR, but these binding sites contain only two heptamers and overlap the-35 promoter boxes of their target genes (Li et al., 2009; He et al., 2010).

## Biotechnological application of SARPs

*Streptomyces* species are considered as the workhorse to produce valuable secondary metabolites (Alam et al., 2022). In recent decades, many strategies have been developed to increase the productivity of these hosts, and rewiring regulatory networks from *Streptomyces* is a powerful and effective approach for yield improvement (Xia et al., 2020; Bu et al., 2021; Sharma et al., 2021). Genetic manipulation of SARPs, the most common activators in *Streptomyces*, has been widely used to promote the production of secondary metabolites in recent decades. As listed in Table 3, the overexpression of *fdmR*, *milR3*, *pieR* and *polR* has been employed to increase fredericamycin production in *S. griseus*, milbemycin production in *S. bingchenggensis*, piericidin production in *S. piomogeues* and polyoxin production in *S. cacaoi* subsp. *asoensis*, respectively (Chen et al., 2008; Li et al., 2009, 2019; Yan et al., 2022). Moreover, promoter strength also plays a critical role in the overexpression of SARP-encoding genes (Table 3). Overexpression of *sanG* with a constitutive promoter led to the overproduction of nikkomycin in *S. ansochromogenes* (He et al., 2010). Overproduction of oxytetracycline has been achieved by introducing an extra copy of *otcR* under the control of the constitutive SF14 promoter in *S. rimosus* (Yin et al., 2015). A similar strategy has also been used to overproduce bafilomycin by overexpressing *bafG* in *S. lohii*, mithramycin by overexpressing *mtmR* in *S. argillaceus* and nigericin by overexpressing *nigR* in *S. malaysiensis* (Flórez et al., 2015; Li et al., 2021; Wei et al., 2022). Moreover, fine-tuning the expression of SARP-encoding genes together with other biosynthetic or regulatory genes can greatly facilitate secondary metabolite production (Table 3). Cooverexpression of *chlF2* and its cotranscribed type II thioesterase-encoding gene *chlK* effectively increased chlorothricin production by 840% in comparison to that of the wild-type strain (Li et al., 2020). Co-overexpression of *bulZ* and the GBL synthetase-encoding gene *bulS2* under a strong promoter in *S. tsukubaensis* improved tacrolimus production by 67.4% compared to that in the wild-type strain (Ma et al., 2018). Overexpression of *srcmRI* and disruption of the PadR-like repressor-encoding gene *srcmRII* led to 750% increased chromomycin production in *Streptomyces roseiscleroticus* (Sun et al., 2018). In our latest research, cooverexpression of *nanR1* and *nanR2* under a strong constitutive promoter in the *milR3* deletion mutant of *S. bingchenggensis* resulted in a 4,500% improvement in the production of nanchangmycin (Yan et al., 2024).

**Figure 2:**
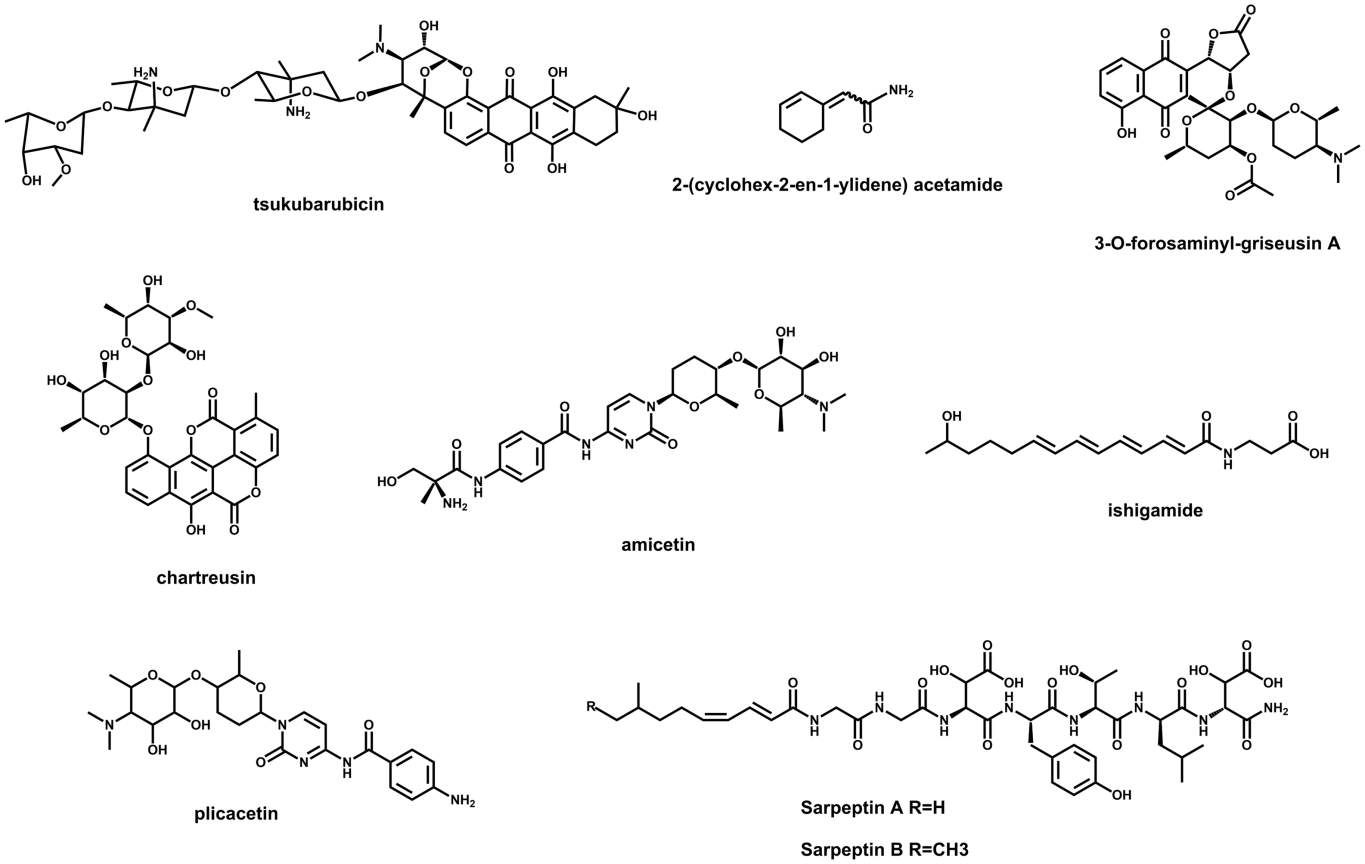
Chemical structures of compounds activated by overexpressing SARPs.

*Streptomyces* species are still promising treasure troves for the discovery of new secondary metabolites due to the abundance of BGCs in their genomes (Lee et al., 2020; Alam et al., 2022). However, a majority of BGCs are silent or weakly expressed under standard laboratory conditions (Misaki et al., 2022). Feasible ways to unlock these cryptic genetic resources are potential strategies for finding new natural products. As the most abundant activators, SARPs are suitable targets to be engineered for the activation of silent BGCs. The chemical structures of several compounds, which were discovered by this method, are illustrated in Figure 2. Cooverexpression of *actII*-*ORF4* from *S. coelicolor* and *aur1PR3* from *Streptomyces aureofaciens* in *Streptomyces* sp. TÜ102 resulted in the activation of the chartreusin BGC (Mingyar et al., 2021). Similarly, simultaneous overexpression of *actII*-*ORF4*, *griR*, *aur1PR3*, *papR2* and *redD* in *Streptomyces* sp. CA-256286 led to the activation of a silent type II PKS gene cluster for griseusin biosynthesis (Beck et al., 2021). Both the prodigiosin BGC in *S. lividans* and the amicetin/plicacetin BGC in *Streptomyces* sp. SHP22-7 were activated by overexpressing *papR2* under the control of a constitutive promoter (Krause et al., 2020). In *Streptomyces* sp. KO-7888, a cryptic NRPS gene cluster was activated by overexpressing the cluster containing the SARP-encoding gene *speR*, which led to the production of two new lipopeptides, sarpeptins A and B (Koomsiri et al., 2019). Additionally, a cryptic ahbamycin gene cluster of *Streptomyces argillaceus* was activated by constitutive overexpression of its cluster-situated SARP-encoding gene (Ye et al., 2023). In addition, the overexpression of *tsuR1*, a putative uncharacterized SARP-encoding gene, led to the discovery of the antitumour antibiotic tsukubarubicin in *S. tsukubaensis* (Wu et al., 2021). Similar strategies have been used to activate the production of a novel amide-containing polyene in *Streptomyces* sp. MSC090213JE08 and a novel cyclohexene-containing enamide in *S. rochei* (Du et al., 2016; Misaki et al., 2022). As mentioned above, SARP-dependent activation is an effective strategy for discovering novel bioactive natural products in *Streptomyces*.

## Conclusions and perspectives

**Figure 3:**
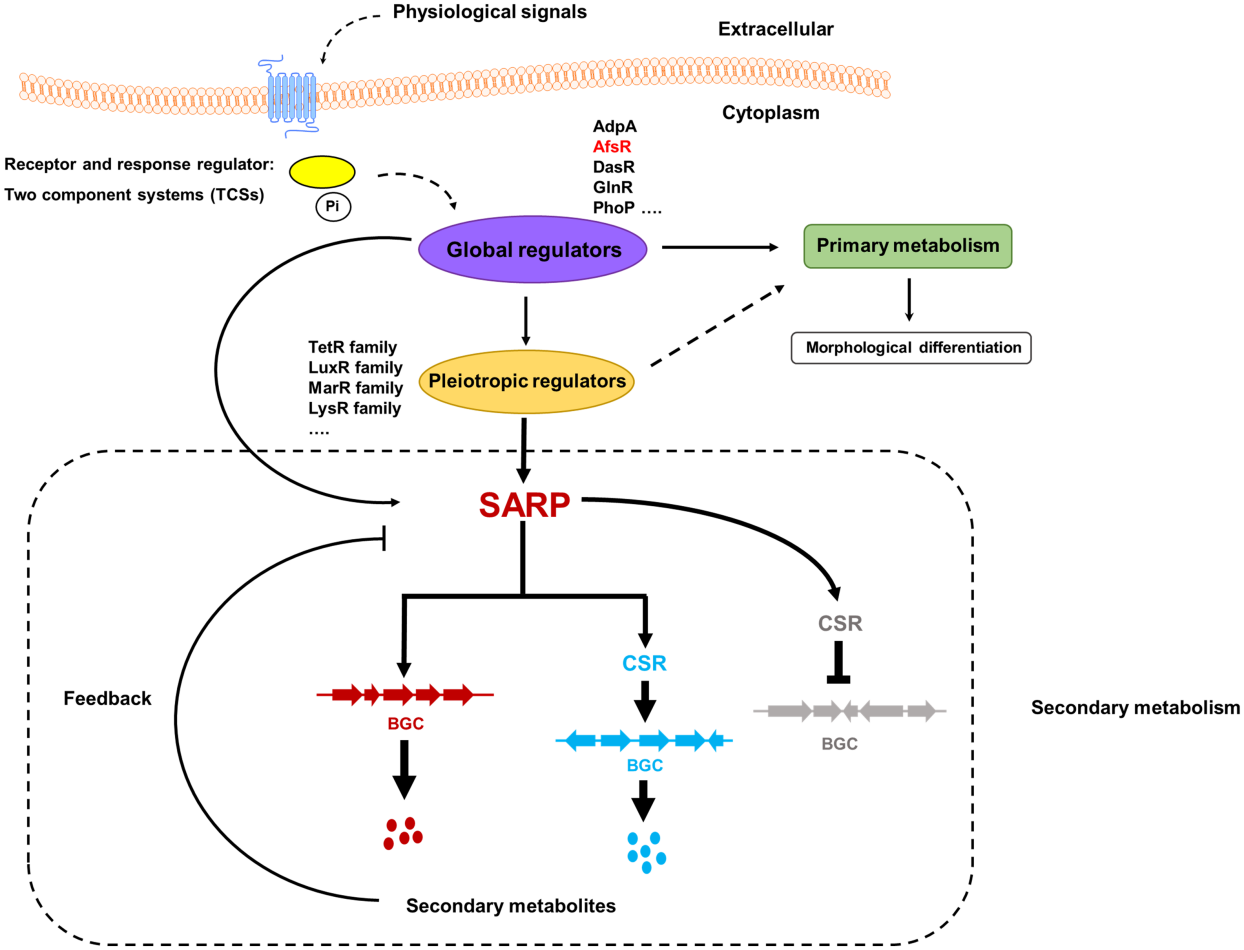
Schematic diagram of the regulatory cascades of SARP family regulators in *Streptomyces*. Two-component systems (TCSs), which consist of a histidine kinase and a response regulator, are the predominant signal transduction pathways involved in the regulation of secondary metabolism. The blue pillars in the cytomembrane indicate a histidine kinase that can sense some physiological signals. The yellow oval indicates a response regulator that is activated by phosphorylation from a signal-triggered histidine kinase. Pi indicates the phosphoric acid group. The known global regulators include AdpA, AfsR, DasR, GlnR, and PhoP. The pleiotropic regulators usually come from different families, such as the TetR family, LuxR family, MarR family, and LysR family. The red and blue dots indicate the cognate secondary metabolites produced by BGCs labeled in red and blue, respectively. The black arrows/dotted arrows indicate a positive effect. The black perpendicular lines indicate negative effects. The regulatory cascades in the dashed rectangle are associated with secondary metabolism.

SARPs, as bottom-level regulators, usually function as cluster-situated activators of secondary metabolism in *Streptomyces*. With extensive studies, the pleiotropic role of SARPs has been recognized. SARPs can also regulate the biosynthesis of multiple secondary metabolites by directly activating the transcription of cognate BGCs and cluster-situated activator-encoding genes in other BGCs. Notably, SARPs negatively affect the biosynthesis of secondary metabolites by activating the transcription of a cluster-situated repressor-encoding gene (Figure 3).

Interestingly, the medium-sized SARP Atr32 serves as a negative regulator of atratumycin biosynthesis in *S. atratus*. In addition to *atr32*, the atratumycin BGC also contains two LuxR family regulator-encoding genes (*atr1* and *atr2*) and two ABC transporter-encoding genes (*atr29* and *atr30*), which play positive roles in atratumycin production (Yang et al., 2019). However, the regulatory relationship between Atr32 and other genes in the atratumycin BGC is unclear. Additionally, when Atr32 performs its negative regulatory function, the role of its NB-ARC domain is unknown. Further elucidation of the repressive regulatory mechanism of Atr32 will enrich the knowledge of SARPs in *Streptomyces*.

The SARP global regulator AfsR is highly conserved and widely distributed in most *Streptomyces* species, and its regulatory mechanism has provided a model for transcriptional activation by SARPs (Table 1) (Tanaka et al., 2007). AfsR interacts with DNA-binding sites in a dimer formation and recruits RNA polymerase (RNAP) to form a DNA-AfsR-RNAP complex that binds to the-10 element of target promoters. Its ATPase domain is essential for conformational changes in the closed complex between AfsR and RNAP to a transcriptionally competent open complex. As mentioned above, SARPs activate the transcription of BGCs in direct or indirect ways. However, how small SARPs without ATPase recruit and interact with RNA polymerase for transcriptional activation is still unknown. With the development of AI-assisted 3D structure elucidation, the elucidation of the mechanism of action and sequence recognition of SARPs will be greatly promoted in the future (Chatonnet et al., 2023).

As mentioned above, the small SARP MilR3/KelR is a pleiotropic regulator that affects the biosynthesis of at least three different secondary metabolites in *S. bingchenggensis* (Yan et al., 2024). Additionally, many putative MilR3/KelR homologs are widely distributed among actinobacteria and likely play similar roles in secondary metabolism (Yan et al., 2022). The cluster-situated activators CpkO and CpkN were identified to form the CpkO–CpkN regulatory cascade for coelimycin biosynthesis in *S. coelicolor*. Furthermore, multi-omics data have shown that these activators also likely regulate the stress response, dormancy and biosynthesis of other specialized metabolites (Bednarz et al., 2021). The mechanism of action of this subgroup of SARPs needs to be extensively investigated in future studies.

Secondary metabolites and/or their biosynthetic intermediates commonly function as feedback or feedforward autoregulators to dynamically modulate their production. This phenomenon is mostly known for TetR family regulators due to their ligand binding domains (Cuthbertson and Nodwell, 2013; Kong et al., 2019). Interestingly, some SARPs, such as NosP in *S. actuosus*, can also respond to secondary metabolites and their intermediates (Li et al., 2018). Recently, the global regulator AfsR in *S. coelicolor* was shown to play an important role in the effect of the artificial elicitor ARC2 (Calvelo et al., 2021). This suggested that some signaling compounds were probably needed for the activation effect of AfsR. Further investigation of the small molecules involved in SARP signal transduction could promote the discovery of new compounds generated by elicitor feeding.

Genetic engineering of SARPs has been proven to be a practical strategy for improving the yield of secondary metabolites and discovering novel bioactive natural products in *Streptomyces*. To date, many more small SARPs have been engineered to enhance the production of secondary metabolites (Table 3). It is obvious that the majority of medium and large SARPs have still not been well investigated (Table 1). Further elucidation of the regulatory mechanisms of SARPs would greatly improve the comprehensive understanding of the mechanism of action of the SARP repertoire, which will be beneficial for developing versatile strategies to enhance final products and unlock novel cryptic BGCs. Increasing the body of knowledge on the diversity of recognition sites and chemical sensing feedback of SARPs will be beneficial for generating programmable signal amplification systems mediated by SARPs, which can facilitate the development of intelligent biomanufacturing in the near future.

## Author contributions

YY: Funding acquisition, Writing – original draft, Writing – review & editing. HX: Funding acquisition, Writing – review & editing.
